# Development and large-scale validation of the Watch Walk wrist-worn digital gait biomarkers

**DOI:** 10.1038/s41598-022-20327-z

**Published:** 2022-10-10

**Authors:** Lloyd L. Y. Chan, Tiffany C. M. Choi, Stephen R. Lord, Matthew A. Brodie

**Affiliations:** 1 Falls, Balance and Injury Research Centre, Neuroscience Research Australia, 139 Baker Street, Randwick, NSW 2031 Australia; 2 School of Population Health, University of New South Wales, Kensington, NSW Australia; 3 School of Health Sciences, Caritas Institute of Higher Education, 2 Chui Ling Lane, New Territories, Tseung Kwan O, Hong Kong China; 4 Graduate School of Biomedical Engineering, University of New South Wales, Samuels Building (F25), Kensington Campus, Kensington, Sydney, NSW 2052 Australia

**Keywords:** Predictive markers, Biomedical engineering

## Abstract

Digital gait biomarkers (including walking speed) indicate functional decline and predict hospitalization and mortality. However, waist or lower-limb devices often used are not designed for continuous life-long use. While wrist devices are ubiquitous and many large research repositories include wrist-sensor data, widely accepted and validated digital gait biomarkers derived from wrist-worn accelerometers are not available yet. Here we describe the development of advanced signal processing algorithms that extract digital gait biomarkers from wrist-worn devices and validation using 1-week data from 78,822 UK Biobank participants. Our gait biomarkers demonstrate good test–retest-reliability, strong agreement with electronic walkway measurements of gait speed and self-reported pace and significantly discriminate individuals with poor self-reported health. With the almost universal uptake of smart-watches, our algorithms offer a new approach to remotely monitor life-long population level walking speed, quality, quantity and distribution, evaluate disease progression, predict risk of adverse events and provide digital gait endpoints for clinical trials.

## Introduction

The use of technology to quantify daily walking activity can provide important indicators of individual and population health^1^. Digital gait biomarkers are quantitative measures of gait derived from wearable device data. As a gait-focused subset of digital biomarkers and mobility outcomes^2,3^, they can be remotely acquired and may provide complementary information to clinical gait assessments^4^. Digital gait biomarkers may be associated with functional status^5^ and general health^6^; and are predictive of functional decline, hospitalization^7^ and mortality^8^.

Previous studies have demonstrated that wearable devices positioned on the lower back or lower limbs can provide valid and reliable digital gait biomarkers. However, their placement on these body regions is awkward which limits user acceptability and compliance^9,10^. In contrast, wrist-worn devices, including smart watches, have superior acceptance and are approaching almost universal uptake. Wrist-worn acceleration data have been acquired in large longitudinal studies, including the UK Biobank^11^, NHANES^12^ and Newcastle85 + studies^13^. For example, the UK Biobank includes 1-week activity data acquired from the AX3 wrist-worn tri-axial accelerometer in 103,578 people in its repository of health data^11^. While measures of physical activity levels^11^ and activity types^14^ have been obtained, the extraction of digital gait biomarkers from these studies has not yet been undertaken.

This omission is likely due to several technical challenges in extracting digital gait biomarkers using a wrist-worn device. Wrist-worn devices are located far from the wearer’s centre of mass and subject to arm movements which increase measurement noise with resultant lower precision and reliability^15,16^. In consequence, conventional digital gait biomarker extraction techniques such as signal peak detection and integration of acceleration with zero-velocity updates can be hampered by large changes in orientation and independent movement of the arms. In fact, the research conducted to date aimed at extracting digital gait biomarkers from wrist-worn devices has mostly been restricted to constrained walks on treadmills and set-length walkways; with resultant algorithms likely inappropriate for more complex walking activities in real-world environments^17,18^. Two studies have used wrist-worn sensors (including barometers and accelerometers) to estimate walking speed and cadence^19,20^. While both studies reported promising results, their inclusion of only healthy volunteers and moderate sample sizes (n ≤ 30 participants) may limit the generalizability of their findings to broader populations. Unsurprisingly, consensus on which digital gait biomarkers are best for remote assessments has yet to be reached^2^.

Clearly, valid and reliable digital gait biomarkers that can be extracted from a wrist-worn device would be valuable for a range of health objectives. We, therefore, aimed to meet this need by conducting a two-stage development and validation study.

In the first stage, 101 participants (19–81 years of age) wore the UK Biobank wrist senor and were recorded while performing a structured mobility routine in free-living settings and then walking and running across an instrumented electronic walkway in our laboratories. We developed (a) the activity classification models using the synchronised video recordings, and subsequently (b) the digital gait biomarker extraction algorithms (including walking speed and cadence) using the instrumented walkway measurements of the instructed walks and runs as ground truth.

In stage two, the convergent validity of the digital gait biomarkers in relation to self-reported walking speed and self-rated health and their test–retest reliability were determined in 78,822 participants from the UK Biobank cohort.

## Results

**Figure 1:**
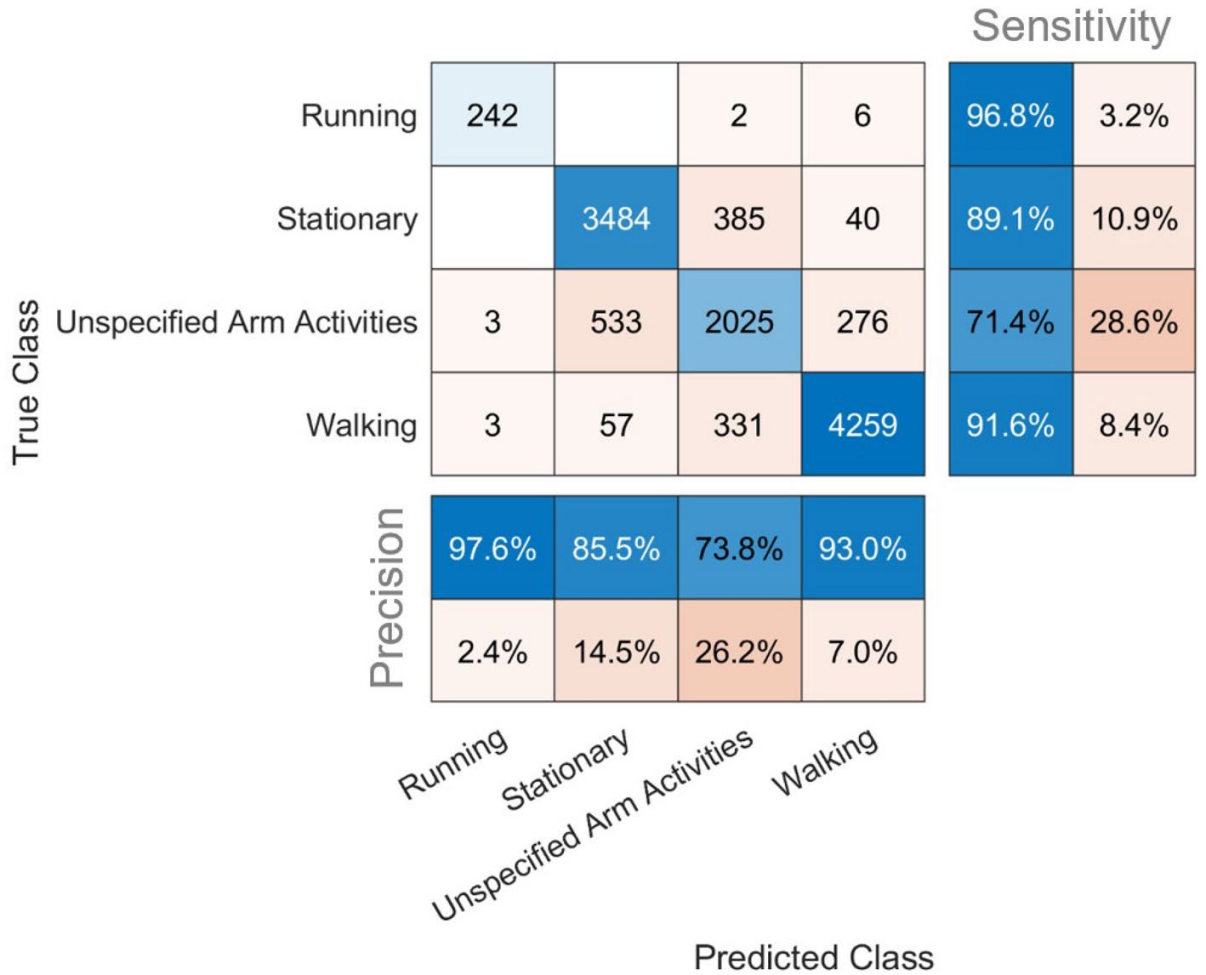
Confusion matrix of stage 1 classification. N = 101 with 11,646 4-s windows. The blue column on the far right of these matrices displays the percentage of correctly identified windows over all the windows that actually belong to that category (i.e. sensitivity). The blue column at the bottom of the matrices represents the percentage of correctly identified windows over all windows that were predicted to be of that category (i.e. precision).

A Support Vector Machine (SVM) classification algorithm was trained for identifying walking bouts from all daily-life activities. A total of 11,646 4-s windows (660 min of free-living recording, 1487 structured walks and 249 structured runs from 101 test participants) were included in the training and validation sets and were classified into Walking, Running, Stationary or Unspecified arm activities. Performance of the classifier was evaluated using a confusion matrix (Fig. 1 and Supplementary Fig. S3).

The walking activity class had a sensitivity of 92% and a precision of 93%; the running activity class had a sensitivity of 97% and a precision of 98%; the stationary activity class had a sensitivity of 89% and precision of 86%; and the unspecified arm activities class had a sensitivity of 71% and precision of 74%.

**Figure 2:**
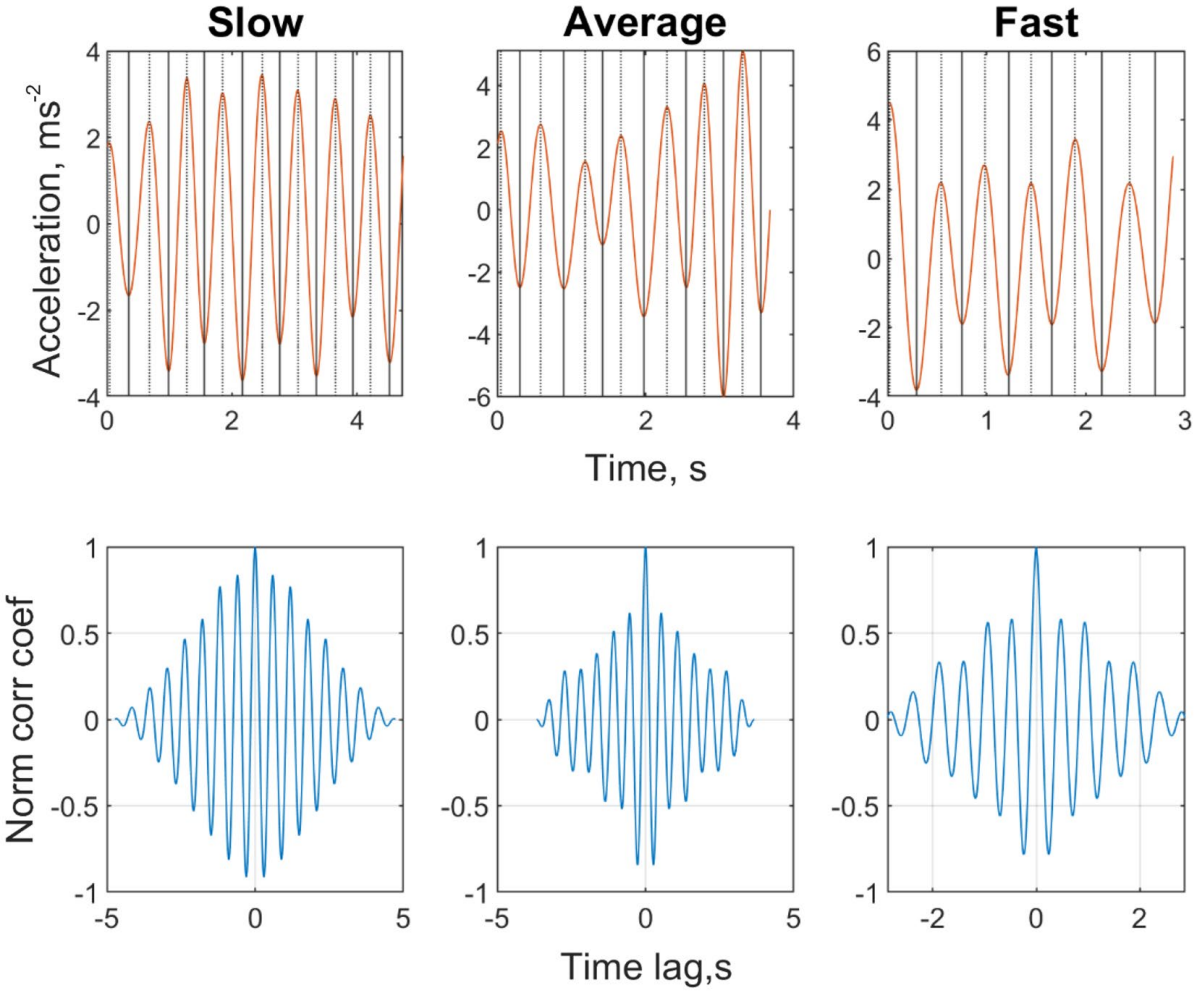
An example of the time-series of the static noise-removed bandpass-filtered Euclidean norm of the acceleration vectors and autocorrelation functions for walking with arm-swing at slow, usual and fast paces.

**Figure 3:**
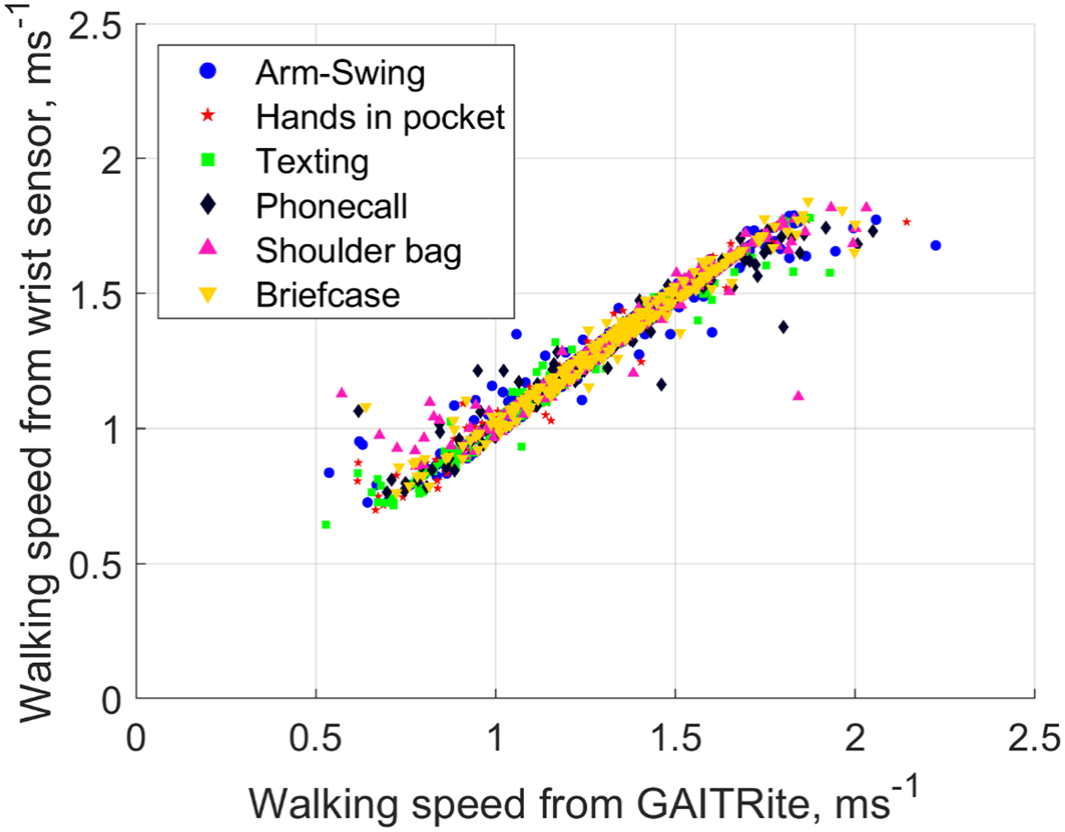
Relationship between wrist sensor and electronic walkway measured walking speed. N = 101 with 1487 walks. Each individual dot represents an individual data point. Combined data from six walking conditions.

Examples of the time-series and autocorrelation function for walking with arm-swing at slow, average and fast paces are presented in Fig. 2. Table 1 presents the accuracy of sensor-based step time and walking speed when compared with the electronic walkway measures. The mean absolute percentage error (MAPE) of step time for the walking conditions ranged between 1.2% and 4.8%, and the MAPE of sensor-based walking speed for the walking conditions ranged between 3.0 and 4.4%. Figure 3 presents the scatterplot for the relationship between walking speed measured by the wrist sensor and the electronic walkway.

**Table 1:** Comparison of spatiotemporal gait parameters assessed by the wrist-worn sensors and the electronic walkway (n = 101 with 1487 walks).

Arm movement pattern while walking	GAITRite Steptime (s)	Sensor-measured step time	GAITRite walking speed (ms^−1^)	Sensor-measured walking speed
	Mean ± SD	Mean absolute percentage error ± SD(%)	Mean ± SD	Mean absolute percentage error ± SD(%)
Arm-SWING	0.54 ± 0.07	4.8 ± 12.4	1.32 ± 0.29	4.4 ± 6.4
Hands in pocket	0.55 ± 0.07	1.4 ± 2.8	1.31 ± 0.26	3.1 ± 3.8
Texting	0.56 ± 0.08	1.2 ± 2.2	1.20 ± 0.27	3.4 ± 3.8
Phonecall	0.55 ± 0.13	1.7 ± 5.5	1.31 ± 0.26	3.4 ± 5.7
Shoulder bag	0.54 ± 0.06	1.4 ± 3.5	1.34 ± 0.25	3.0 ± 4.9
Briefcase	0.53 ± 0.07	2.4 ± 8.1	1.37 ± 0.27	3.8 ± 8.4

*ms*^−*1*^ m per second, *s* second, *SD* standard deviation.

**Figure 4:**
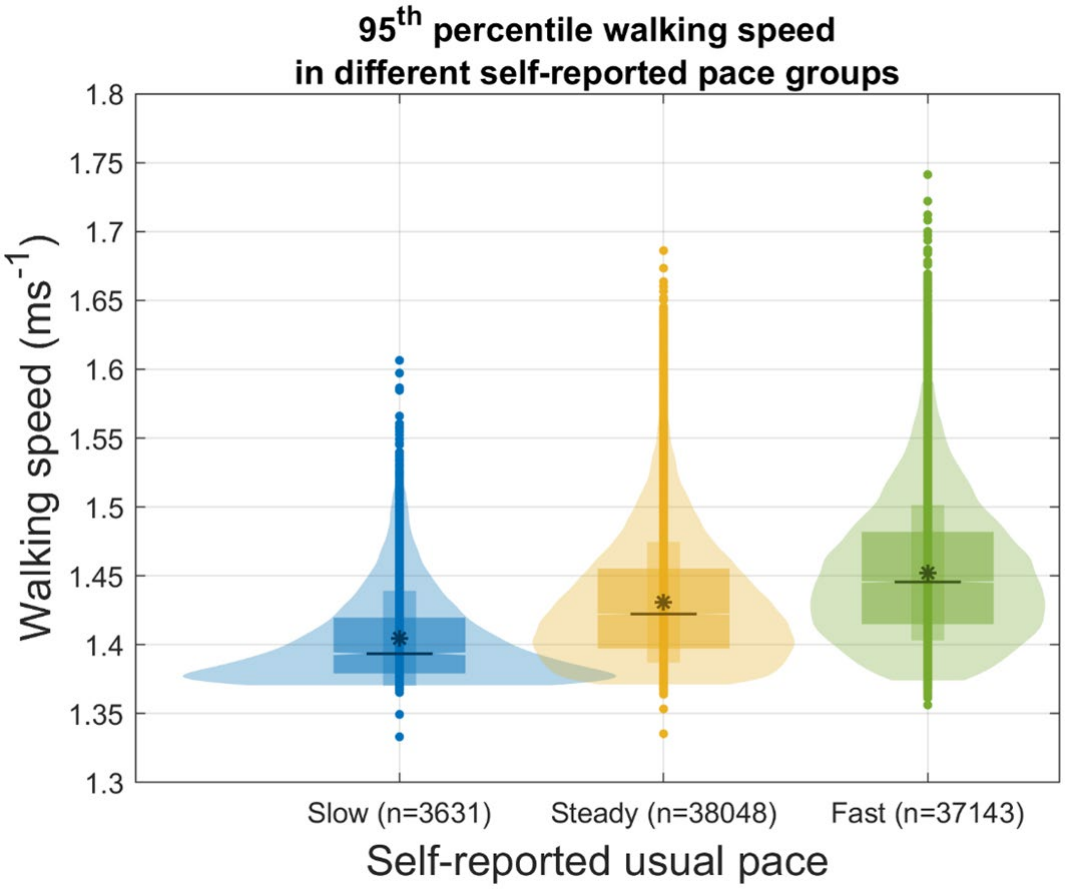
Maxmial walking speed for people who reported slow, steady and brisk walking paces. N = 78,822. Individual dots at the upper and lower extreme, raw data outliers. Widths of the violin plots, kemel desnsities; Top and bottom of the violin plots, 1st and 99th percentiles; Top and bottom of the narrower boxs, mean ± standard deviation; Top and bottom of the widers box, 1st and 3rd Quartile; Notches of the wider box, 95% confidence intervals of the population median; Black lines in the middle of the boxs, group medians; The asterisks in the middle of the boxes, group means.

**Figure 5:**
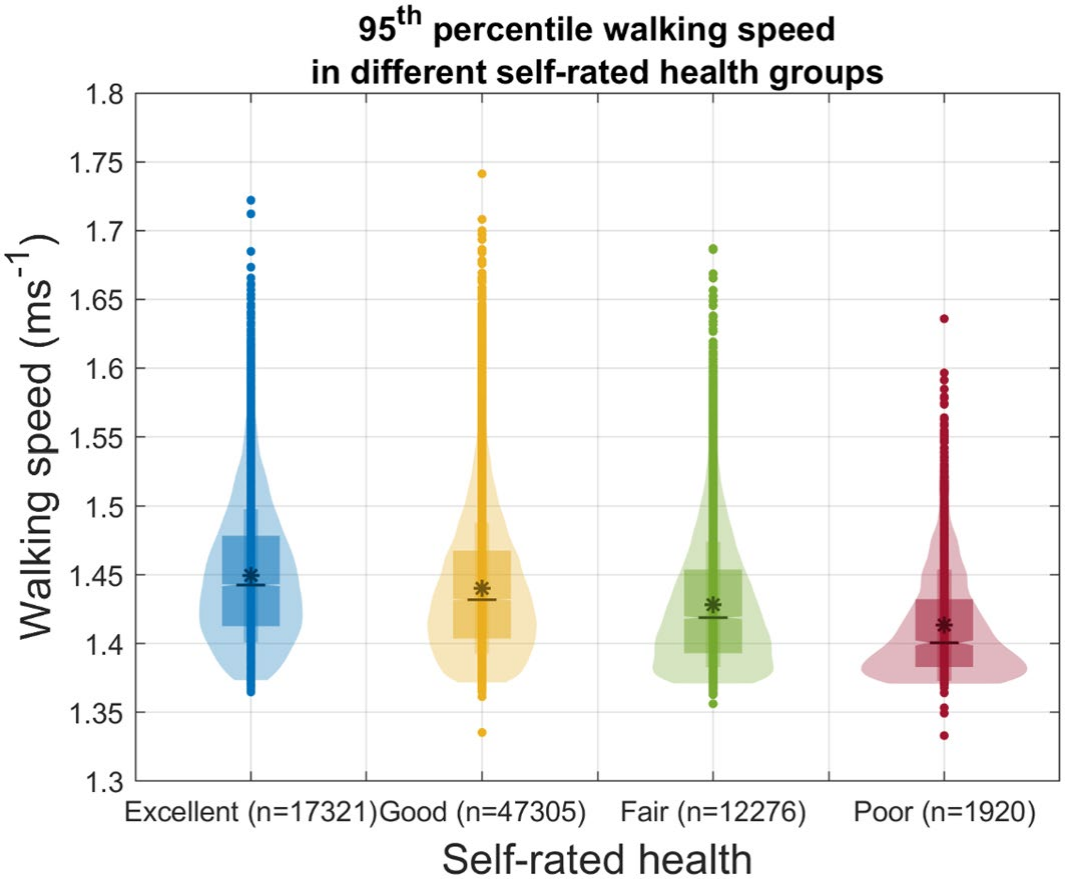
Maximal walking speed for people who reported poor, fair and good and excellent health. N = 78,822. Individual dots at the upper and lower extreme, raw data outliers. Widths of the violin plots, kemel desnsities; Top and bottom of the violin plots, 1st and 99th percentiles; Top and bottom of the narrower boxs, mean ± standard deviation; Top and bottom of the widers box, 1st and 3rd Quartile; Notches of the wider box, 95% confidence intervals of the population median; Black lines in the middle of the boxs, group medians; The asterisks in the middle of the boxes, group means. *ms*^−*1*^ metre per second.

Table 2 presents the comparison of maximal walking speed between UK Biobank participants who reported routinely walking at a slow, steady or brisk walking pace. The maximal walking speed differed significantly between participants who usually walked at a slow pace (median: 1.39 ms^1^, inter-quartile range: 1.38–1.42 ms^1^), steady pace (median: 1.42 ms^1^, inter-quartile range: 1.40–1.46 ms^1^), and brisk pace (median: 1.45 ms^1^, inter-quartile range: 1.42–1.48 ms^1^), (Fig. 4). All extracted digital gait biomarkers also differed significantly between individuals with different self-reported health levels (Table 3, Fig. 5) Post-hoc comparison between groups are presented in Supplementary Table S3. Finally, most extracted digital gait biomarkers, except longest walk duration and stride regularity (ICC 0.66 and 0.68 respectively), demonstrated good test–retest reliability (ICCs ranging from 0.71 to 0.89).

**Table 2:** Maximal walking speed stratified by self-reported walking pace and self-rated health (n = 78,822).

	Maximal walking speed (arm-swing), ms^−1^		Kruskal–Wallis test		
	Median	IQR	df	Chi-square	P-value
**Pace**			78,821	6643.7	< 0.001
Slow	1.39	1.38–1.42			
Steady	1.42	1.40–1.46			
Brisk	1.45	1.42–1.48			
**Self-reported health**			78,821	2380.7	< 0.001
Excellent	1.44	1.41–1.48			
Good	1.43	1.40–1.47			
Fair	1.42	1.39–1.45			
Poor	1.40	1.38–1.43			

P-values of the Dunn post-hoc test between each group are all < 0.001.

*IQR* Inter-quartile range, *ms*^−*1*^ m per second.

**Table 3:** Summary statistics and test–retest reliability of digital gait biomarkers (n = 78,822).

Self-rated Health	Excellent (N = 17,321)	Good (N = 47,305)	Fair (N = 12,276)	Poor (N = 1920)	Total (N = 78,822)	p-value	ICC
Demographics							
Female, n (%)	9778 (56.5%)	26,051 (55.1%)	5988 (48.8%)	943 (49.1%)	42,760 (54.2%)	< .0001^1^	
Age	57.0 (49.0, 62.0)	58.0 (50.0, 63.0)	57.0 (50.0, 62.0)	56.0 (50.0, 61.0)	57.0 (50.0, 62.0)	< .0001^2^	
**Gait quantity and its distribution**							
Steps per day	8671.4 (3273.21)	8111.6 (3261.81)	7089.0 (3275.40)	5666.1 (3209.29)	8015.7 (3321.01)	< .0001^3^	0.83
Longest walk duration [s]	266.9 (172.6, 399.2)	244.0 (152.0, 373.7)	202.7 (121.6, 324.6)	140.7 (77.3, 249.3)	240.6 (148.0, 370.7)	< .0001^2^	0.66
Arm-swing proportion [%]	79.3 (71.6, 86.1)	80.1 (72.5, 87.0)	81.4 (73.6, 88.2)	83.0 (75.3, 89.1)	80.2 (72.5, 87.1)	< .0001^2^	0.89
Hands in pocket proportion [%]	4.2 (3.0, 5.9)	4.0 (2.8, 5.6)	3.7 (2.6, 5.3)	3.4 (2.3, 4.8)	4.0 (2.8, 5.6)	< .0001^2^	0.78
Texting proportion [%]	8.3 (5.5, 11.6)	8.2 (5.4, 11.5)	8.0 (5.2, 11.4)	8.3 (5.2, 11.8)	8.2 (5.4, 11.5)	< .0001^2^	0.88
Phone-call proportion [%]	0.6 (0.2, 1.3)	0.5 (0.2, 1.3)	0.5 (0.2, 1.3)	0.5 (0.2, 1.3)	0.5 (0.2, 1.3)	< .0001^2^	0.78
Shoulder-bag proportion [%]	2.5 (1.1, 5.0)	2.2 (1.0, 4.5)	1.8 (0.8, 3.9)	1.3 (0.6, 2.9)	2.2 (1.0, 4.5)	< .0001^2^	0.82
Briefcase proportion [%]	2.2 (1.0, 4.2)	2.0 (0.9, 3.9)	1.6 (0.7, 3.4)	1.1 (0.4, 2.7)	2.0 (0.9, 3.8)	< .0001^2^	0.81
Step-walk gradient*100	− 111.6 (23.13)	− 113.9 (23.99)	− 116.6 (25.94)	− 122.3 (28.59)	− 114.0 (24.33)	< .0001^3^	0.76
Walks ≤ 8 s [%]	54.3 (10.16)	55.3 (10.47)	56.7 (11.22)	60.5 (12.38)	55.4 (10.63)	< .0001^3^	0.80
Walks ≤ 60 s [%]	94.9 (89.7, 98.0)	95.4 (90.3, 98.2)	96.0 (91.3, 98.6)	97.1 (92.8, 99.4)	95.4 (90.4, 98.3)	< .0001^2^	0.75
**Gait speed**							
Median (usual) [ms^−1^]	1.33 (1.32, 135)	1.33 (1.31, 1.35)	1.32 (1.30, 1.34)	1.32 (1.30, 1.34)	1.33 (1.31, 1.35)	< .0001^2^	0.86
95th percentile(maximal) [ms^−1^]	1.44 (1.41, 1.48)	1.43 (1.40, 1.47)	1.42 (1.39, 1.45)	1.40 (1.38, 1.43)	1.43 (1.40, 1.47)	< .0001^2^	0.85
**Gait quality**							
Cadence median [spm]	105.9 (5.91)	105.0 (5.76)	103.8 (5.67)	102.4 (5.53)	105.0 (5.82)	< .0001^3^	0.85
Cadence IQR [spm]	22.0 (4.42)	21.7 (4.43)	21.6 (4.59)	22.4 (5.15)	21.8 (4.48)	< .0001^3^	0.77
Mode of step-time variability[s]	0.028 (0.018, 0.041)	0.031 (0.02, 0.045)	0.036 (0.022, 0.051)	0.044 (0.028, 0.061)	0.031 (0.020, 0.045)	< .0001^2^	0.77
8-step HR (arm-swing)	1.39 (0.41)	1.36 (0.42)	1.31 (0.45)	1.17 (0.49)	1.36 (0.43)	< .0001^3^	0.77
Step regularity (arm-swing) [%]	66.4 (55.3, 76.0)	64.6 (53.2, 74.9)	61.5 (49.3, 72.8)	55.5 (43.1, 69.1)	64.4 (52.8, 74.8)	< .0001^2^	0.71
Stride regularity (arm-swing) [%]	54.7 (42.6, 65.3)	53.1 (40.5, 64.6)	50.8 (37.5, 63.6)	44.3 (31.1, 59.4)	53.0 (40.2, 64.5)	< .0001^2^	0.68

*HR* harmonic ratio, *ICC* intraclass correlation, *IQR* interquartile range, *ms*^−*1*^ m per second, *s* second, *spm* steps per minute.

^1^Count and proportion with Chi-Square p-value.

^2^Median and Inter-quartile range with Kruskal–Wallis p-value;

^3^Mean and standard deviation with ANOVA F-test p-value.

## Discussion

The main aims of this study were to develop and validate digital gait biomarkers derived from a wrist-worn device using both laboratory-assessed and real-world data. We found our digital gait biomarkers demonstrated good test–retest reliability, strongly agreed with electronic walkway measurements of gait speed and self-reported pace and significantly discriminated between individuals with poor and good self-reported health. The algorithms were readily applied in the large UK Biobank database that collected 7-day wrist-sensor data indicating the good utility of these measures.

The Watch Walk method presented in this paper enables the retrieval of digital gait biomarkers that summarise walking speed, gait quality, walking patterns and the statistical distributions of gait measures in daily life. These digital gait biomarkers, commonly assessed through sensors located at inconvenient attachment sites (i.e. ankle and lower back), have been proven to accurately predict adverse health events^21,22^ and used as surrogate endpoints in pharmaceutical trials^23^. Watch Walk advances the range and depth of measures obtained from wrist-worn accelerometer measures, which have predominantly featured step-count^24^, vector magnitude as a proxy of physical activity intensity^11^ and time spent in sleep, physical activity and sedentary behaviours^14^. Watch walk builds on previous advances using wrist-worn sensors to estimate walking speed and cadence^19,20^ through enhanced generalizability and new applications in large-scale activity monitoring. Considering that wrist-worn accelerometers are widely available, either incorporated in commercially-available smart-watches or measurement tools in large longitudinal studies, our new method offers a practical tool to remotely monitor multiple aspects of mobility, which has been considered as the sixth vital sign^6^, in a reliable, valid and cost-effective way.

Our study findings build on previous work undertaken in this field in several ways. First, despite daily-life gait parameters varying from day-to-day^25^, our results showed that most of the digital gait biomarkers computed as daily averages over seven-days have good test–retest reliability. This supports the continued use of seven-day wrist-worn accelerometry measures that have high compliance in older adults^9^ and are commonly used in physical activity research^26^. Second, our classification accuracy for walking activities was above 91% which compares favourably with a previous activity classification algorithm proposed for the UK Biobank dataset (70% in the CAPTURE24 study)^14^. It is likely our higher accuracy was due to the use of 4-s window frames that better reflect the short walking bouts undertaken in daily life^27^. Third, compared to previous studies that estimated walking speed/distance with wrist worn accelerometers^17,18^, our development and technical validation study involved a substantially larger sample (101 participants), a wider age range of participants (19–81 years of age) and included more diverse walking activities and different hand positions while walking; all factors that provide greater external generalizability and reduce over-feeding bias. Finally, The Watch Walk method was developed with a hardware agnostic approach and the data format required (seven-day 100 Hz tri-axial wrist acceleration) has been widely utilized. Hence, it is applicable to not only the UK Biobank dataset, but also to other healthcare databases such as NHANES, as well as future studies using commercially available smartwatches.

We also acknowledge certain limitations. First, our measure of sensor-based walking speed was validated with an electronic walkway in a laboratory setting and daily-life activity classification was based on simulated, as opposed to real life, day-to-day activities. Second, it was not practical to use body-camera recordings to validate our activity classification schema in real-life due to the short timeframes and wide dispersion of the activity capture windows. Third, the digital gait biomarkers were not validated in participants who use walking aids, so such walks may have been missed. Finally, walking speed accuracy was lower for walks slower than 0.7 ms^−1^ and faster than 1.8 ms^−1^, i.e. beyond 2 standard deviations of mean gait-speed of older adults^28^. Further studies are required to refine walking speed estimations at these extremes.

Future studies should investigate the clinical validity of these new digital gait biomarkers by: (1) developing normative values to provide a reference base to identify mobility impairments in clinical populations; (2) assessing their predictive capabilities with regard to important clinical outcomes such as falls and fall-related injuries, frailty, cognitive impairment and mortality; and (3) using them to monitor the progression of chronic conditions and evaluate the effectiveness of interventions. The use of these practical and objective measurements of daily-life mobility performance may also assist in the early detection of mobility decline to enable early interventions to maximise health and wellbeing.

## Methods

### Stage 1: development and initial evaluation of an activity classification schema and Watch Walk digital gait biomarker algorithms

#### Participants

One hundred and one participants aged 19–81 years (mean 47 ± 18(SD)) (67% female) were recruited from two study sites through volunteer databases (HC190949) and online advertisements from 2020 to 2021: 51 in Sydney, Australia and 50 in Hong Kong, China. The participants’ mean height was 1.67 m (± 0.1 m) and their mean weight was 66 kg (± 14 kg). The methods were performed in accordance with relevant guidelines and regulations and approved by the Human Research Ethics Committees at the University of New South Wales (HC200839) and Caritas Institute of Higher Education (HRE210124).

All participants gave written informed consent prior to inclusion.

#### Assessments

Participants wore an AX3 data logger (Axtivity Limited, Newcastle upon Tyne, UK) on their dominant wrist, configured according to UK Biobank’s data collection protocol and were video-recorded while they undertook a series of mobility tasks. The AX3 data logger is a compact device (23 × 33 × 8 mm) weighing 11 g that contains a tri-axial logging accelerometer. Acceleration data were sampled at 100 Hz with a range of ± 8 gravitational acceleration units (g).

Participants first walked and ran on a 5.7 m electronic walkway (GAITRite, CIR Systems Inc. Franklin NJ, USA) at three paces (usual, slower than usual and faster than usual) for seven conditions (walking with arm swing, walking with hands in pockets, walking while texting, walking with a mobile phone held to the ear, walking while carrying a bag over the shoulder, walking while carrying a briefcase and jogging). Gait speed (metre per second), step time (second), step length (metre) and the standard deviation (SD) of step times were extracted using the GAITrite software.

Participants then performed a series of semi-structured daily-life activities in a set order in areas where they frequently encountered other people. No specific instructions were given as to how to perform the tasks, which included: sitting down and standing up from a chair; lying down and getting up from a mattress; walking along a corridor; taking an elevator; walking up and down stairs; writing, typing, reading a book and tying shoelaces while seated; and washing hands and rinsing a cup in the sink while standing. Wearable sensor data were synchronised with the video data and manually annotated (e.g. marking the start and end-points of a walk with arm swing) by a trained exercise physiologist.

#### Pre-processing of data

A sample level Euclidean norm from the x/y/z axes acceleration vectors was obtained^29^. Static noise was subsequently removed through subtracting the average signal amplitude over 60 s from the resulting Euclidean norm^29^. A fifth order Butterworth low pass filter with a cut-off frequency of 20 Hz was applied to remove machine noise. A low pass filter with a frequency passband of 0.25 and 2.5 Hz was also applied to the Euclidean norm to facilitate acceleration signal peak detection^30^. Subsequently, non-wear episodes and sleep period time windows were removed. Non-wear episodes were defined as consecutive stationary episodes that exceed 50 minutes with a standard deviation of 13 milli-gravity units or less^11^. Sleep period time windows were identified using the method proposed by van Hees and colleagues^31^. Acceleration vectors were separated into non-overlapping 4-s windows (rationale for this window size provided in supplementary material). A vector of 54-dimensional features was extracted from 99 features through backward selection of feature importance (Supplementary Table S1 and Supplementary Fig. S2). This included the mean, standard deviation, 25th, 50th, 75th percentile of the static-block-removed and crude Euclidean norms of the acceleration signal respectively. It also included the correlation coefficients between the local x, y, and z accelerations and the normalized autocorrelation coefficient, the ratio between the 1st-2nd and 1st-3rd autocorrelation values and time-lag.

#### Activity classification

The activity classification algorithms were trained and validated using the Matlab Statistics and Machine Learning Toolbox version 11.6. Support vector machines (SVMs) were used for a multi-class classification of activities, as it has been demonstrated to be highly accurate and robust in activity recognition^32^. Initially, six activity categories were trained: (1) walking with arm-swing; (2) other complex walking; (3) running; (4) stationary (which includes windows that captured travelling in vehicles); (5) unspecified arm activities while standing/ sitting; and (6) unspecified arm activities while walking. The second refined classification separated windows under “Other complex walking” into the five annotated sub-categories: (a) walking with hands in pockets; (b) walking while texting; (c) walking with a mobile phone held to the ear; d) Walking while carrying a bag over the shoulder; and (e) walking while carrying a briefcase/grocery bag. Activity classification was trained with ten-fold cross validation with data partitioning at individual-level. This is arranged to avoid over-estimation of prediction accuracy from intra-class correlation. The activity categories are described in Supplementary Table S2.

#### Extraction of the Watch Walk digital gait biomarkers

##### Gait quantity and its distribution

**Figure 6:**
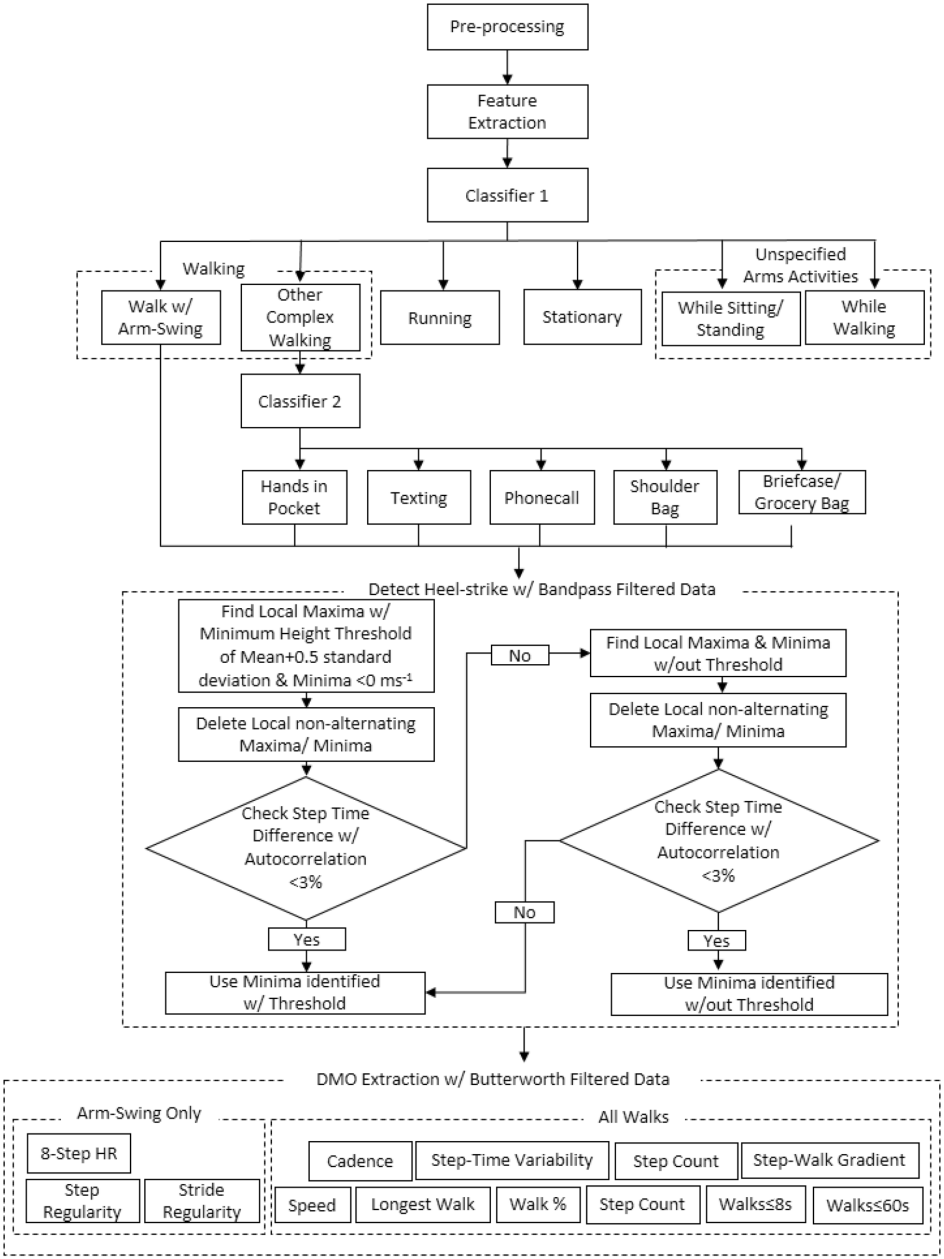
Hierarchical framework of digital gait biomarker extraction (Stage 1).

In periods classified as walking, steps were detected with bandpass filtered acceleration local maxima and local minima. Local maxima and local minima were checked to ensure they were alternating, aligned with autocorrelation-estimated step time and were higher/lower than the adaptive thresholds respectively^29^. Details of the step-detection process is summarised in Fig. 6. Total step count was defined as the total number of steps detected per day and the longest walking bout was defined as the duration of the largest number of consecutive walking windows. The proportion of duration in walking with arm-swing to the total duration of all forms of walking were extracted. The distribution between number of walks and the steps per walk were obtained by fitting a linear model to the log–log transformed data. A steeper slope (β1) represents that more short walks and fewer longer walks were performed. Gait quantity was also quantified through the cumulative exposure of walking durations (equation below)^33^.

$$X_{i} = \frac{{\Sigma d\,\,for\,\,d \le d_{i} }}{\Sigma d\,}*100\%$$

The percentage of walks of less than (1) 7 s and (2) 60 s were extracted. Total minutes of running per day was obtained through the activity classification process.

##### Gait speed

Average walking speed in each 4-s window was estimated through fitting a Medium Gaussian SVM regression model (10-fold validation) with (1) height of the participant^34^, (2) interquartile range and (3) median of the Static-block-removed Euclidean norm of acceleration signal, (4) mean of crude Euclidean norm of acceleration signal, (5) mean step time within the window and correlation coefficients between acceleration signal in (6) x- /y- axes and (7) x- /z- axes. The median, 95th percentile and interquartile range of walking speed in a 24-h period were extracted. Median walking speed represents the usual walking speed in daily life. The 95th percentile of walking speed represents the maximal walking speed in daily life with outliers excluded, which reflects an individual’s optimal gait performance better than medium values^35^.

##### Gait quality

Walks were further regrouped into 8-step episodes. Walks longer than the cut-off were separated into smaller parts. For example, a walk with 18 steps was separated into 1st to 8th steps, 9th to 16th steps and with the 17th and 18th steps truncated. Cadence was obtained through measuring the time required for an 8-step episode. The standard deviation of step times was used to quantify step-time variability. A log-normal model was fitted to the distribution to extract the mode (equation below).

$$Mode = e^{{\mu - \sigma^{2} }}$$

Eight-step harmonic ratios were defined as the repeating patterns in the Euclidean norm acceleration (stabilising peaks) over the incomplete patterns (destabilising troughs) implemented with a fast Fourier transform^36^. The 8-step harmonic ratio has demonstrated good test–retest reliability (ICC = 0.72) and perform better in identifying fall risk when compared to the traditional (2-step) harmonic ratio. Step and strike regularity were extracted through autocorrelation. The first and second peaks of autocorrelation values indicate correlation between steps and between strides respectively and were subsequently normalized through dividing by auto-correlation value at zero time-lag. Hence the resulting parameters vary only from − 1 to + 1.

Further details of the gait quality biomarkers have been reported by Brodie et al.^33^ in their study on remote monitoring with pendent devices.

### Statistical analysis

Pre-processing, algorithm development and parameter extraction were completed in MATLAB, version R2019b. The hierarchical framework of the extraction process is presented in Fig. 6. The accuracy of the activity classification algorithms was examined with ten-fold validation, using the annotated class of the walking and running trials on the electronic walkway, the semi-structured daily-life activity routine and vehicle passenger episodes as the ground truth activities. Sensitivity and precision of each class were presented along with confusion matrixes. The criterion validity of the Walk Watch step time and walking speed biomarkers were tested against the corresponding measurements from the electronic walkway and reported as mean absolute percentage error (MAPE).

### Stage 2: test–retest reliability and convergent validity of the digital gait biomarkers with respect to self-reported walking pace and health

#### Participants

Participants for stage 2 comprised 78,822 participants from the UK biobank. Participants were instructed to wear an AX3 data logger over their dominant wrist for seven days in 2013. They aged 46–77 years (Median 64, IQR 57–69) (56% female). Ethical approval for UKBiobank data transfer and analysis was obtained from the NHS National Research Ethics Service (Ref 11/NW/0382). Participant flow is presented in Supplementary Fig. S1.

#### Data quality and exclusions

Accelerometry data were excluded if considered to be of low quality by the UK Biobank accelerometer working group due to: (1) the data were collected with accelerometers that were poorly calibrated; (2) the accelerometry data were of an abnormal size; and/or (3) the data collection period contained a daylight savings transition. In addition, we used only participant data with 24-h sensor wear-time for five or more days with at least one walking bout and complete self-reported walking pace and self-rated health data.

#### Statistical analysis

Test–retest reliability of the Watch Walk digital gait biomarkers were examined with intraclass correlation coefficients (2-way random effects, absolute agreement, mean of multiple measurements) for seven consecutive days. The Watch Walk digital gait biomarkers were contrasted between participants self-rated health status using the Kruskal–Wallis test and Dunn post-hoc test for non-parametric continuously scaled data; ANOVA and Tukey post-hoc test for parametric continuously scaled data; and chi square test for contingency tables for categorical data. The maximal walking speed was compared among participants who reported slow, average and brisk walking paces with Kruskal–Wallis test, with post hoc comparisons performed with the Dunn post-hoc test.

## Supplementary Information

Supplementary Information.

## Data Availability

Publicly available data from the UK Biobank data are available through procedures described at http://www.ukbiobank.ac.uk/using-the-resource/. Digital Gait Biomarkers data will be returned to UK Biobank within 6 months after publication of this paper.
