# Author Correction: Development and large-scale validation of the Watch Walk wrist-worn digital gait biomarkers

**DOI:** 10.1038/s41598-023-28191-1

**Published:** 2023-01-20

**Authors:** Lloyd L. Y. Chan, Tiffany C. M. Choi, Stephen R. Lord, Matthew A. Brodie

**Affiliations:** 1 Falls, Balance and Injury Research Centre, Neuroscience Research Australia, 139 Baker Street, Randwick, NSW 2031 Australia; 2 School of Population Health, University of New South Wales, Kensington, NSW Australia; 3 School of Health Sciences, Caritas Institute of Higher Education, 2 Chui Ling Lane, New Territories, Tseung Kwan O, Hong Kong, China; 4 Graduate School of Biomedical Engineering, University of New South Wales, Samuels Building (F25), Kensington Campus, Kensington, Sydney, NSW 2052 Australia

Correction to: *Scientific Reports* 10.1038/s41598-022-20327-z, published online 10 October 2022

The original version of this Article contained errors in Table 3, where the values for “Mode of step-time variability” and “8-step HR (arm-swing)” under “Gait Quality” were incorrect. The correct and incorrect values appear below.

Incorrect:

**Table:** 

Self-rated Health	Excellent (N = 17,321)	Good (N = 47,305)	Fair (N = 12,276)	Poor (N = 1920)	Total (N = 78,822)	p-value	ICC
**Gait Quality**							
Mode of step-time variability	2.8 (1.8, 4.1)	3.1 (2.0, 4.5)	3.6 (2.2, 5.1)	4.4 (2.8, 6.1)	3.1 (2.0, 4.5)	< .0001^2^	0.77
8-step HR (arm-swing)	13.9 (4.07)	13.6 (4.23)	13.1 (4.51)	11.7 (4.91)	13.6 (4.27)	< .0001^3^	0.77

Correct:

**Table:** 

Self-rated Health	Excellent (N = 17,321)	Good (N = 47,305)	Fair (N = 12,276)	Poor (N = 1920)	Total (N = 78,822)	p-value	ICC
**Gait Quality**							
Mode of step-time variability [s]	0.028 (0.018, 0.041)	0.031 (0.02, 0.045)	0.036 (0.022, 0.051)	0.044 (0.028, 0.061)	0.031 (0.020, 0.045)	< .0001^2^	0.77
8-step HR (arm-swing)	1.39 (0.41)	1.36 (0.42)	1.31 (0.45)	1.17 (0.49)	1.36 (0.43)	< .0001^3^	0.77

The original Article has been corrected.

