# Supplementary material for: Development and large-scale validation of the Watch Walk wrist-worn digital gait biomarkers
Source: Sci Rep. 2022 Oct 10;12:16211. doi: 10.1038/s41598-022-20327-z (PMC9551062; doi:10.1038/s41598-022-20327-z)
Supplement: Supplementary file 1 — Supplementary Information. [file 41598_2022_20327_MOESM1_ESM.docx]

**Supplementary Table 1.** List of machine learning features generated for selection

| Feature No. | Name (unit) and description | Rank |
| --- | --- | --- |
| One window before the current window | | |
| 1 | Wavelet coefficient in Static-block-removed Euclidean norm of acceleration signal  (scales 1 to 12) | 93 |
| 2 | Wavelet coefficient in Static-block-removed Euclidean norm of acceleration signal  (scales 12 to 22 | 83 |
| 3 | Wavelet coefficient in Static-block-removed Euclidean norm of acceleration signal  (scales 22 to 37 | 60 |
| 4 | Wavelet coefficient in Static-block-removed Euclidean norm of acceleration signal  (scales 37 to 57 | 57 |
| 5 | Wavelet coefficient in Static-block-removed Euclidean norm of acceleration signal  (scales 57 to 85 | 56 |
| 6 | Wavelet coefficient in Static-block-removed Euclidean norm of acceleration signal  (scales 85 to 115 | 53 |
| 7 | Wavelet coefficient in Static-block-removed Euclidean norm of acceleration signal  (scales 115 to 165 | 52 |
| 8 | Wavelet coefficient in Static-block-removed Euclidean norm of acceleration signal  (scales 165 to 245 | 51 |
| 9 | Mean of Static-block-removed Euclidean norm of acceleration signal | 50 |
| 10 | Standard deviation of Static-block-removed Euclidean norm of acceleration signal | 49 |
| **11** | **Minimum of Static-block-removed Euclidean norm of acceleration signal** | **46** |
| 12 | Maximum of Static-block-removed Euclidean norm of acceleration signal | 58 |
| 13 | 25^th^ percentile of Static-block-removed Euclidean norm of acceleration signal | 48 |
| **14** | **Median of Static-block-removed Euclidean norm of acceleration signal** | **27** |
| 15 | 75^th^ percentile of Static-block-removed Euclidean norm of acceleration signal | 80 |
| **16** | **Correlation coefficient between acceleration signal in x- and y- axes** | **18** |
| **17** | **Correlation coefficient between acceleration signal in x- and z- axes** | **43** |
| **18** | **Correlation coefficient between acceleration signal in y- and z- axes** | **14** |
| 19 | Mean of crude vector magnitude | 82 |
| 20 | Standard deviation of crude vector magnitude | 84 |
| **21** | **Minimum of crude vector magnitude** | **16** |
| 22 | Maximum crude vector magnitude | 59 |
| **23** | **25^th^ percentile of crude vector magnitude** | **15** |
| **24** | **Median of crude vector magnitude** | **24** |
| **25** | **75^th^ percentile of crude vector magnitude** | **17** |
| 26 | Number of peaks in autocorrelation | 81 |
| 27 | Normalised autocorrelation coefficient | 47 |
| 28 | Autocorrelation coefficient | 65 |
| 29 | Ratio between 1^st^ and 2^nd^ autocorrelation coefficient | 75 |
| **30** | **Ratio between 1^st^ and 2^nd^ autocorrelation time-lag** | **19** |
| 31 | Ratio between 1^st^ and 3^rd^ autocorrelation coefficient | 89 |
| 32 | Ratio between 1^st^ and 3^rd^ autocorrelation time-lag | 79 |
| 33 | Time-lag of 1^st^ autocorrelation | 85 |
| The current window | | |
| 34 | Wavelet coefficient in Static-block-removed Euclidean norm of acceleration signal  (scales 1 to 12) | 76 |
| **35** | **Wavelet coefficient in Static-block-removed Euclidean norm of acceleration signal**  **(scales 12 to 22)** | **23** |
| 36 | Wavelet coefficient in Static-block-removed Euclidean norm of acceleration signal  (scales 22 to 37) | 86 |
| **37** | **Wavelet coefficient in Static-block-removed Euclidean norm of acceleration signal**  **(scales 37 to 57)** | **13** |
| 38 | Wavelet coefficient in Static-block-removed Euclidean norm of acceleration signal  (scales 57 to 85) | 90 |
| **39** | **Wavelet coefficient in Static-block-removed Euclidean norm of acceleration signal**  **(scales 85 to 115)** | **42** |
| 40 | Wavelet coefficient in Static-block-removed Euclidean norm of acceleration signal  (scales 115 to 165) | 54 |
| **41** | **Wavelet coefficient in Static-block-removed Euclidean norm of acceleration signal**  **(scales 165 to 245)** | **10** |
| **42** | **Mean of Static-block-removed Euclidean norm of acceleration signal** | **9** |
| **43** | **Standard deviation of Static-block-removed Euclidean norm of acceleration signal** | **25** |
| 44 | Minimum of Static-block-removed Euclidean norm of acceleration signal | 98 |
| **45** | **Maximum of Static-block-removed Euclidean norm of acceleration signal** | **40** |
| **46** | **25^th^ percentile of Static-block-removed Euclidean norm of acceleration signal** | **30** |
| **47** | **Median of Static-block-removed Euclidean norm of acceleration signal** | **38** |
| **48** | **75^th^ percentile of Static-block-removed Euclidean norm of acceleration signal** | **32** |
| 49 | Correlation coefficient between acceleration signal in x- and y- axes | 96 |
| 50 | Correlation coefficient between acceleration signal in x- and z- axes | 91 |
| **51** | **Correlation coefficient between acceleration signal in y- and z- axes** | **20** |
| **52** | **Mean of crude vector magnitude** | **36** |
| **53** | **Standard deviation of crude vector magnitude** | **44** |
| 54 | Minimum of crude vector magnitude | 67 |
| 55 | Maximum crude vector magnitude | 70 |
| 56 | 25^th^ percentile of crude vector magnitude | 63 |
| **57** | **Median of crude vector magnitude** | **34** |
| 58 | 75^th^ percentile of crude vector magnitude | 72 |
| 59 | Number of peaks in autocorrelation | 87 |
| **60** | **Normalised autocorrelation coefficient** | **37** |
| **61** | **Autocorrelation coefficient** | **3** |
| **62** | **Ratio between 1^st^ and 2^nd^ autocorrelation coefficient** | **5** |
| **63** | **Ratio between 1^st^ and 2^nd^ autocorrelation time-lag** | **45** |
| **64** | **Ratio between 1^st^ and 3^rd^ autocorrelation coefficient** | **1** |
| **65** | **Ratio between 1^st^ and 3^rd^ autocorrelation time-lag** | **39** |
| **66** | **Time-lag of 1^st^ autocorrelation** | **4** |
| One window after the current window | | |
| **67** | **Wavelet coefficient in Static-block-removed Euclidean norm of acceleration signal**  **(scales 1 to 12)** | **7** |
| 68 | Wavelet coefficient in Static-block-removed Euclidean norm of acceleration signal  (scales 12 to 22) | 55 |
| 69 | Wavelet coefficient in Static-block-removed Euclidean norm of acceleration signal  (scales 22 to 37) | 71 |
| **70** | **Wavelet coefficient in Static-block-removed Euclidean norm of acceleration signal**  **(scales 37 to 57)** | **2** |
| **71** | **Wavelet coefficient in Static-block-removed Euclidean norm of acceleration signal**  **(scales 57 to 85)** | **21** |
| 72 | Wavelet coefficient in Static-block-removed Euclidean norm of acceleration signal  (scales 85 to 115) | 62 |
| **73** | **Wavelet coefficient in Static-block-removed Euclidean norm of acceleration signal**  **(scales 115 to 165)** | **11** |
| **74** | **Wavelet coefficient in Static-block-removed Euclidean norm of acceleration signal**  **(scales 165 to 245)** | **6** |
| **75** | **Mean of Static-block-removed Euclidean norm of acceleration signal** | **35** |
| **76** | **Standard deviation of Static-block-removed Euclidean norm of acceleration signal** | **29** |
| 77 | Minimum of Static-block-removed Euclidean norm of acceleration signal | 73 |
| 78 | Maximum of Static-block-removed Euclidean norm of acceleration signal | 77 |
| **79** | **25^th^ percentile of Static-block-removed Euclidean norm of acceleration signal** | **41** |
| **80** | **Median of Static-block-removed Euclidean norm of acceleration signal** | **8** |
| 81 | 75^th^ percentile of Static-block-removed Euclidean norm of acceleration signal | 74 |
| 82 | Correlation coefficient between acceleration signal in x- and y- axes | 88 |
| **83** | **Correlation coefficient between acceleration signal in x- and z- axes** | **31** |
| 84 | Correlation coefficient between acceleration signal in y- and z- axes | 69 |
| **85** | **Mean of crude Euclidean norm of acceleration signal** | **12** |
| 86 | Standard deviation of crude Euclidean norm of acceleration signal | 64 |
| 87 | Minimum of crude Euclidean norm of acceleration signal | 97 |
| **88** | **Maximum crude Euclidean norm of acceleration signal** | **22** |
| 89 | 25^th^ percentile of crude Euclidean norm of acceleration signal | 78 |
| **90** | **Median of crude Euclidean norm of acceleration signal** | **33** |
| **91** | **75^th^ percentile of crude Euclidean norm of acceleration signal** | **28** |
| 92 | Number of peaks in autocorrelation | 68 |
| 93 | Normalised autocorrelation coefficient | 94 |
| 94 | Autocorrelation coefficient | 92 |
| 95 | Ratio between 1^st^ and 2^nd^ autocorrelation coefficient | 95 |
| **96** | **Ratio between 1^st^ and 2^nd^ autocorrelation time-lag** | **26** |
| 97 | Ratio between 1^st^ and 3^rd^ autocorrelation coefficient | 66 |
| 98 | Ratio between 1^st^ and 3^rd^ autocorrelation time-lag | 99 |
| 99 | Time-lag of 1^st^ autocorrelation | 61 |

Bold indicates features included in the activity classification algorithm

**Supplementary Table 2.** List of annotated activity categories

| Activity class | **Category** | **Description** |
| --- | --- | --- |
| Walking | Walking: Arm swing | Refers to walking with natural arm swing |
|  | Other walking patterns | - Walking- Hands in pockets: Refers to walking with the dominant hand in a trousers/ shorts/ skirt/dress pocket - Walking- Texting : Refers to walking while holding a smart phone stably in front of the trunk with the dominant hand - Walking- Phone call: Refers to walking while holding a smart phone stably next to the head with the dominant hand - Walking- Shoulder bag: Refers to walking with the dominant hand rested on or in front of the shoulder - Walking- Briefcase: Refers to walking with the dominant arm straight on the side while carrying a heavy object |
| Running | Running | Refers to any running movement |
| Stationary | Stationary | Refers to standing or sitting with minimal or without upper limb movement |
|  | Vehicle | Refers to standing or sitting with minimal or without upper limb movement on a moving vehicle |
| Unspecified Arms Activities | Unspecified arms activities while sitting/ standing | Refers to a standing or sitting with upper limb movements |
|  | Unspecified arms activities while walking | Refers to walking with independent upper limb movements |

**Supplementary Table 3**: P-values of post-hoc comparison tests between different self-rated health status (n=78822). Respondents were asked to rate their overall health as “Excellent”, “Good”, “Fair” or “Poor”.

|  | **Self-rated Health Status** | | | | | |
| --- | --- | --- | --- | --- | --- | --- |
|  | **Excellent vs Good** | **Excellent vs Fair** | **Excellent vs Poor** | **Good vs Fair** | **Good vs Poor** | **Fair vs Poor** |
| **Demographics** | | | | | | |
| Age^1^ | **<0.001** | **<0.001** | 0.23 | 0.22 | **<0.001** | **<0.001** |
| **Gait quantity and its distribution** | |  |  |  |  |  |
| Steps per day^2^ | **<0.001** | **<0.001** | **<0.001** | **<0.001** | **<0.001** | **<0.001** |
| Longest walk duration^1^ | **<0.001** | **<0.001** | **<0.001** | **<0.001** | **<0.001** | **<0.001** |
| Arm-swing proportion^1^ | **<0.001** | **<0.001** | **<0.001** | **<0.001** | **<0.001** | **<0.001** |
| Hands in pocket proportion^1^ | **<0.001** | **<0.001** | **<0.001** | **<0.001** | **<0.001** | **<0.001** |
| Texting proportion^1^ | 0.34 | **<0.001** | 0.94 | **<0.001** | 1.00 | 0.43 |
| Phone-call proportion^1^ | **0.04** | **<0.001** | 0.10 | **<0.001** | 0.53 | 0.99 |
| Shoulder-bag proportion^1^ | **<0.001** | **<0.001** | **<0.001** | **<0.001** | **<0.001** | **<0.001** |
| Briefcase proportion^1^ | **<0.001** | **<0.001** | **<0.001** | **<0.001** | **<0.001** | **<0.001** |
| Step-walk Gradient^2^ | **<0.001** | **<0.001** | **<0.001** | **<0.001** | **<0.001** | **<0.001** |
| Walks≤8s | **<0.001** | **<0.001** | **<0.001** | **<0.001** | **<0.001** | **0.01** |
| Walks≤60s^1^ | **<0.001** | **<0.001** | **<0.001** | **<0.001** | **<0.001** | **<0.001** |
| **Gait Speed (arm-swing)** | | | | | | |
| Median (usual) ^1^ | **<0.001** | **<0.001** | **<0.001** | **<0.001** | **<0.001** | **<0.001** |
| 95th percentile(maximal) ^1^ | **<0.001** | **<0.001** | **<0.001** | **<0.001** | **<0.001** | **<0.001** |
| **Gait Quality** | | | | | | |
| Cadence Median^2^ | **<0.001** | **<0.001** | **<0.001** | **<0.001** | **<0.001** | **<0.001** |
| Cadence IQR ^2^ | **<0.001** | **<0.001** | **<0.001** | **<0.001** | **<0.001** | **<0.001** |
| Mode of step-time variability^1^ | **<0.001** | **<0.001** | **<0.001** | **<0.001** | **<0.001** | **<0.001** |
| 8-step HR ^2^ | **<0.001** | **<0.001** | **<0.001** | **<0.001** | **<0.001** | **<0.001** |
| Step regularity (arm-swing) ^1^ | **<0.001** | **<0.001** | **<0.001** | **0.01** | **<0.001** | **<0.001** |
| Stride regularity (arm-swing) ^1^ | **<0.001** | **<0.001** | **<0.001** | **<0.001** | **<0.001** | **<0.001** |

Bold indicates statistical significance
^1^Dunn post-hoc test
^2^Tukey's honest significance test

**Supplementary Figure 1:** Participant flow chart for Stage II study (n= 103672)
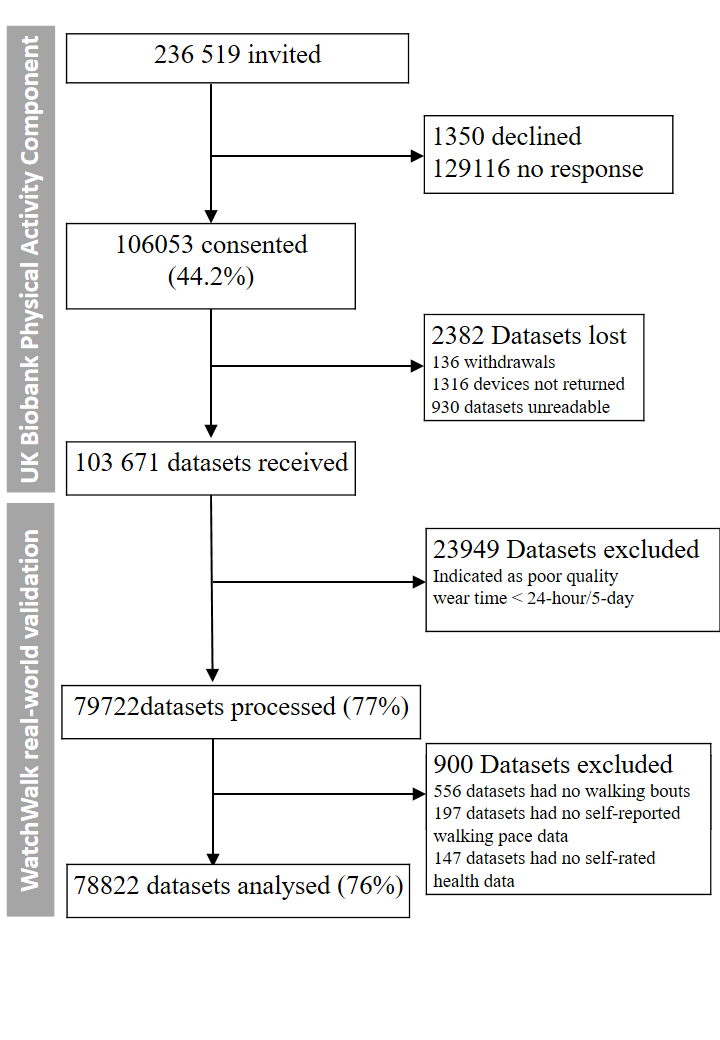


**Supplementary Figure 2:** Accuracy and Cohen’s Kappa of the classification model by the number of features included **
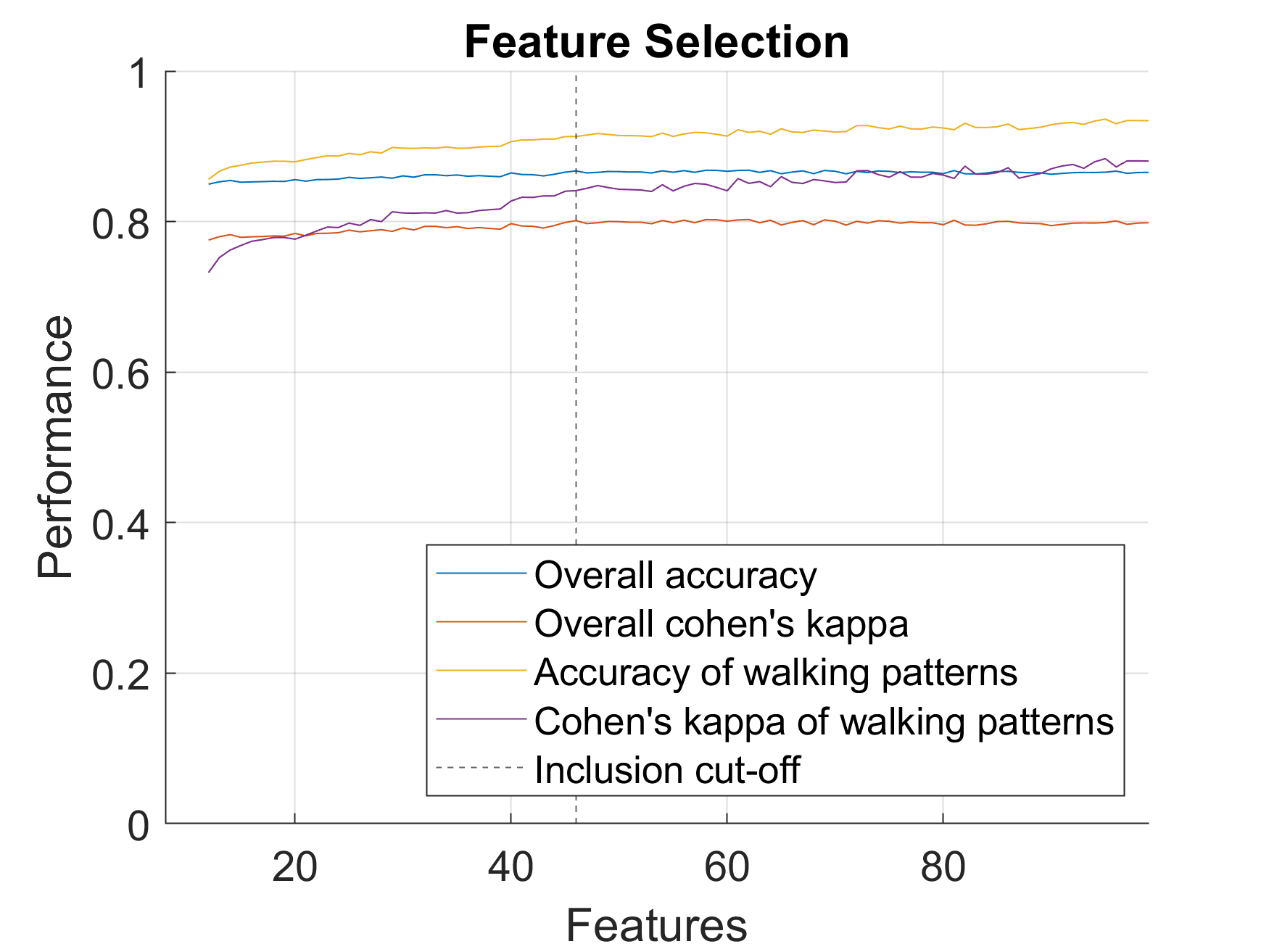
**

**Supplementary Figure 3.** Confusion matrix of stage 2 classification (4,388 windows from both free-living recording and structured walks)
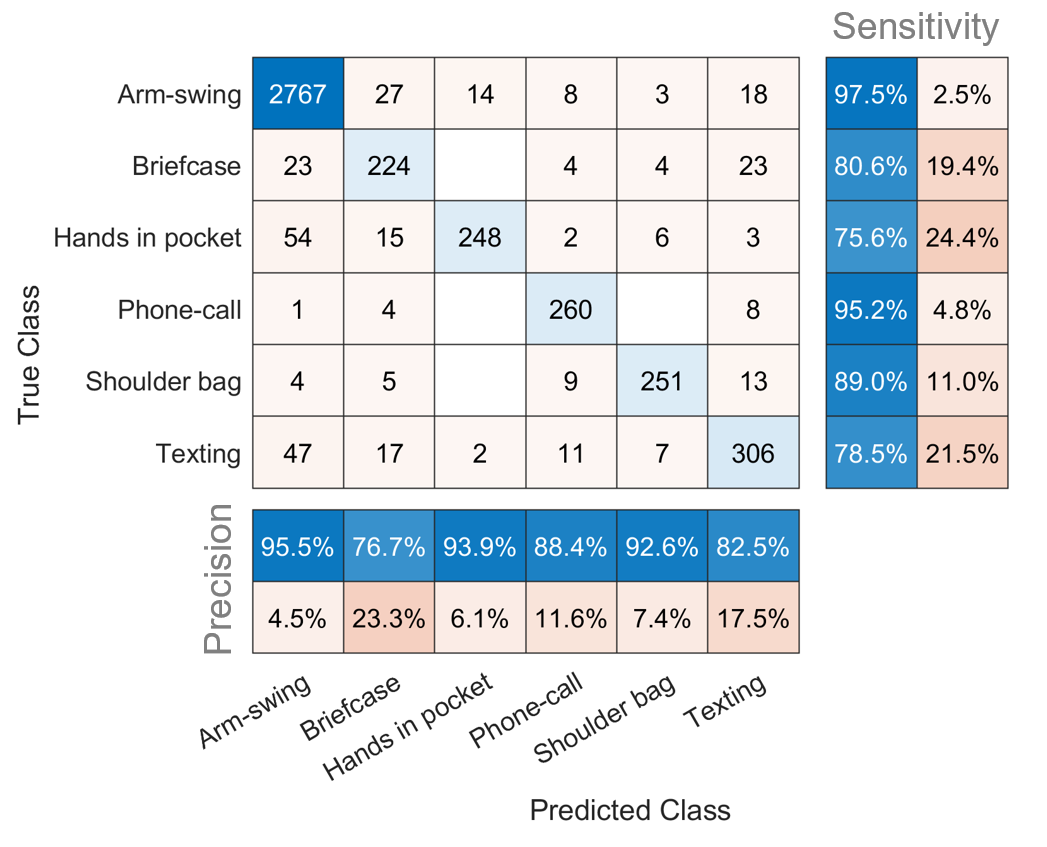


**Supplemetary material: Justification of using 4-second window**

To classify movement into activity classes, data were segmented into 4-second(s) non-overlapping windows. In theory, longer window segments (e.g. >10s) provide more samples and repetitions to be extracted and therefore enhance classifier performance. However, daily activities often occur in short bouts. For instance, while entering a lift, a person stands quietly, walks and then stands quietly again all within10 s. Shorter window segments would better segment these activities and as well as reduced feedback latency and therefore better for providing real-time responses. Mannini et al have compared activity recognition performances of 12.8s, 4s and 2s window segments, and found 4s and 12.8s windows to be similarly accurate (84.2% to 84.7%).^1^ Similarly, Zhang et al reported that 12.8s windows can be reduced to 6.8s windows without significantly compromising classification accuracy^2^.

^1^ Mannini A, Intille SS, Rosenberger M, Sabatini AM, Haskell W. Activity recognition using a single accelerometer placed at the wrist or ankle. Med Sci Sports Exerc. 2013 Nov;45(11):2193-203. doi: 10.1249/MSS.0b013e31829736d6. Erratum in: Med Sci Sports Exerc. 2015 Feb;47(2):448-9. PMID: 23604069; PMCID: PMC3795931.

^2^ Zhang S, Rowlands AV, Murray P, Hurst TL. Physical activity classification using the GENEA wrist-worn accelerometer. Med Sci Sports Exerc. 2012 Apr;44(4):742-8. doi: 10.1249/MSS.0b013e31823bf95c. PMID: 21988935.
